# Sex-specific time trends of long-term graft survival after kidney transplantation – a registry-based study

**DOI:** 10.1080/0886022X.2023.2270078

**Published:** 2023-10-26

**Authors:** Salmir Nasic, Björn Peters, Bernd Stegmayr, Elisabeth Kenne Sarenmalm, Henri Afghahi, Marie Eriksson

**Affiliations:** a Department of Molecular and Clinical Medicine, Institute of Medicine, The Sahlgrenska Academy at University of Gothenburg, Gothenburg, Sweden; b Research, Education, Development and Innovation Department, Skaraborg Hospital, Skövde, Sweden; c Department of Nephrology, Skaraborg Hospital, Skövde, Sweden; d Public Health and Clinical Medicine, Umeå University, Umeå, Sweden; e Institute of Health and Care Sciences, The Sahlgrenska Academy at University of Gothenburg, Gothenburg, Sweden; f Department of Statistics, Umeå School of Business, Economics and Statistics, Umeå University, Umeå, Sweden

**Keywords:** Kidney graft survival, kidney transplants, sex-specific trends

## Abstract

**Background:**

Sex-specific trends over time with respect to kidney graft survival have scarcely been described in earlier studies. The present study aimed to examine whether kidney graft survival differs between women and men over time.

**Methods:**

This study was based on prospectively collected data extracted from a quality registry including all kidney transplant patients between January 1965 and September 2017 at the transplantation center of a university hospital in Sweden. The transplantation center serves a population of approximately 3.5 million inhabitants. Only the first graft for each patient was included in the study resulting in 4698 transplantations from unique patients (37% women, 63% men). Patients were followed-up until graft failure, death, or the end of the study. Death-censored graft survival analysis after kidney transplantation (KT) was performed using Kaplan-Meier analysis with log-rank test, and analysis adjusted for confounders was performed using multivariable Cox regression analysis.

**Results:**

Median age at transplantation was 48 years (quartiles 36–57 years) and was similar for women and men. Graft survival was analyzed separately in four transplantation periods that represented various immunosuppressive regimes (1965-1985, 1986–1995, 1996–2005, and 2006–2017). Sex differences in graft survival varied over time (sex-by-period interaction, *p* = 0.026). During the three first periods, there were no significant sex differences in graft survival. However, during the last period, women had shorter graft survival (*p* = 0.022, hazard ratio (HR) 1.71, 95% confidence interval (CI) 1.1–2.7, adjusted for covariates). Biopsy-proven rejections were more common in women.

**Conclusions:**

In this registry-based study, women had shorter graft survival than men during the last observation period (years 2006–2017).

## Introduction

Graft survival after kidney transplantation has markedly improved over the years [1–3] most likely due to improved immunosuppressive treatment (IST), but in part, may be related to improved knowledge and medical care in general [4]. Multiple factors influence graft loss after kidney transplantation [5], some of which have been well-studied. The importance of matching donor-recipient sex has been described in several studies, but with varying results. Some studies have shown that donor-recipient sex differences do not affect graft survival [6,7] but according to Kwon et al. [8] female donor-to-male recipients had the worst graft survival. Living donor grafts in general entail longer graft survival compared to deceased donor grafts [4,7,9,10]. The impact of donor and recipient age has been described in different studies [11,12]. Graft survival was generally shorter for grafts from older donors, whereas it was longer in older recipients.

In some studies, graft survival did not differ between female and male recipients [6,7,12–14]. However, there are studies showing shorter graft survival in men [15], whereas in other studies, women had shorter graft survival [16–18]. Nonetheless, differences between women and men concerning kidney graft survival over time and those related to different immunosuppressive regimens have been sparsely described.

The aim of the present study was to examine whether kidney graft survival differs over time between women and men transplanted from 1965 to 2017 at a single center representing 3.5 million inhabitants. If differences existed, the secondary aim was to identify the risk factors.

## Material and methods

### Study population and design

In this study, we performed graft survival analysis using registry-based data. Data collected from the quality assessment registry (TIGER) were described in detail in a previous study [19]. The registry included all kidney transplant patients who underwent transplantation between January 1965 and September 2017 at the Sahlgrenska University Hospital in Gothenburg, Sweden. The transplantation center serves a population of approximately 3.5 million inhabitants. In addition, data from a separate biopsy register that included biopsies from 2007 to 2017 were used to explore the associations between biopsy-proven diagnosis and graft survival [19]. Only the first kidney transplant for each patient was used in the analysis, and only adult patients (≥18 years of age) were included in this study.

### Definitions

Graft loss after kidney transplantation (KT) was defined as the start of dialysis or re-transplantation.

Primary renal disease (PRD) as the cause of kidney transplantation was registered for all patients and all diagnoses of PRD were classified into eight main groups (Table 1).

**Table 1.** Patient characteristics for kidney transplanted patients according to sex and time period of transplantation.

	Transplantation period, years													
	1965–1985 (n = 1216)			1986–1995 (n = 1104)			1996-2005 (*n* = 957)			2006–2017 (*n* = 1421)			Total (*n* = 4698)	
	Women *n* = 468	Men *n* = 748	*p*-value	Women *n* = 421	Men *n* = 683	*p*-value	Women *n* = 346	Men *n* = 611	p-value	Women *n* = 507	Men *n* = 914	p-value	Women n = 1742	Men *n* = 2956
*Age, median (IQR)*	43 (33–52)	42 (33–52)	0.628	48 (35–58)	45 (36–54)	0.102	51 (38–59)	49 (38–58)	0.479	51 (40–60)	52 (42–61)	0.040	48 (36–57)	48 (37–57)
*Deceased donor, n (%)*	339 (72.4)	557 (74.5)	0.434	314 (74.6)	510 (74.7)	0.974	236 (68.6)	376 (61.8)	0.036	324 (63.9)	602 (65.9)	0.458	1213 (69.7)	2045 (69.3)
*Primary renal disease* ^*a*^ *,n (%)*														
Glomerulonephritis/ sclerosis	129 (27.6)	346 (46.3)	<0.001	89 (21.1)	235 (34.4)	<0.001	71 (20.5)	177 (29.0)	0.001	89 (17.6)	267 (29.2)	<0.001	378 (21.7)	1025 (34.7)
Pyelonephritis	158 (33.8)	107 (14.3)		74 (17.6)	57 (8.3)		40 (11.6)	36 (5.9)		13 (2.6)	23 (2.5)		285 (16.4)	223 (7.5)
Polycystic kidney disease	59 (12.6)	84 (11.2)		61 (14.5)	88 (12.9)		66 (19.1)	94 (15.4)		116 (22.9)	117 (12.8)		302 (17.3)	383 (13.0)
Renal vascular disease due to hypertension	4 (0.9)	40 (5.3)		10 (2.4)	22 (3.2)		8 (2.3)	19 (3.1)		13 (2.6)	36 (3.9)		35 (2.0)	117 (4.0)
Renal vascular disease (unspecific)	1 (0.2)	2 (0.3)		3 (0.7)	4 (0.6)		2 (0.6)	5 (0.8)		7 (1.4)	9 (1.0)		13 (0.7)	20 (0.7)
Diabetes mellitus	60 (12.8)	98 (13.1)		74 (17.6)	139 (20.4)		42 (12.1)	90 (14.7)		73 (14.4)	150 (16.4)		249 (14.3)	477 (16.1)
Miscellaneous	23 (4.9)	22 (2.9)		67 (15.9)	71 (10.4)		26 (7.5)	25 (4.1)		112 (22.1)	155 (17.0)		228 (13.1)	273 (9.2)
Unknown	34 (7.3)	49 (6.6)		43 (10.2)	67 (9.8)		91 (26.3)	165 (27.0)		84 (16.6)	157 (17.2)		252 (14.5)	438 (14.8)

^a^ Grouping according to ERA-EDTA Registry: ERA-EDTA Registry Annual Report 2019. Amsterdam UMC, location AMC, Department of Medical Informatics, Amsterdam, The Netherlands, 2021.

Unknown: unspecific chronic kidney failure.

The observed time of all transplantations (years 1965–2017) was divided into the following four periods: 1965–1985, 1986–1995, 1996–2005, and 2006–2017 (Table 2). The breakpoints for the four time periods were based on the goal of finding time points for major changes in patterns of immunosuppressive treatment, as well as keeping plausible numbers of patients in each transplantation period.

**Table 2.** Immunosuppressive drugs as maintenance therapy based on protocols registered at discharge after kidney transplantation. Occurrence (%) of each drug. 3500 of 4698 transplanted patients had valid protocols at discharge^a^.

	Transplantation period (years)											
	1965–1985 Predominant Azathioprine			1986–1995 Predominant Cyclosporine			1996–2005 Predominant Cyclosporine/MMF			2006-2017 Predominant MMF/Tacrolimus		
Total number	Women n = 468	Men n = 748	p-value	Women n = 421	Men n = 683	p-value	Women n = 346	Men n = 611	p-value	Women n= 507	Men n = 914	p-value
Valid protocols^b^	n = 264	n = 405		n = 268	n = 455		n = 264	n = 492		n = 482	n = 870	
Azathioprine, n (%)	216 (81.8)	322 (79.5)	0.461	245 (91.4)	431 (94.7)	0.081	73 (27.7)	163 (33.1)	0.121	3 (0.6)	4 (0.5)	0.690
Prednisolon, n (%)	263 (99.6)	402 (99.3)	0.553	268? (100)	453 (99.6)	0.277	261 (98.9)	488 (99.2)	0.658	446 (92.5)	791 (90.9)	0.309
Cyclosporine, n (%)	66 (25.0)	93 (23.0)	0.545	266 (99.3)	453 (99.6)	0.591	209 (79.2)	389 (79.1)	0.974	126 (26.1)	266 (30.6)	0.085
MMF, n (%)	0	0	n.a	0	3 (0.7)	0.183	177 (67.0)	306 (62.2)	0.186	410 (85.1)	700 (80.5)	0.034
Tacrolimus, n (%)	0	0		0	1 (0.2)	n.a	42 (15.9)	80 (16.3)	0.900	298 (61.8)	468 (53.8)	0.004
Sirolimus, n (%)	0	0	n.a	0	0	n.a	8 (3.0)	26 (5.3)	0.154	4 (0.8)	4 (0.5)	0.395
Myfortic, n (%)	0	0	n.a	0	0	n.a	0	0	n.a	61 (12.7)	130 (14.9)	0.248
Tacrolimus depot, n (%)	0	0	n.a	0	0	n.a	0	1 (0.2)	n.a	45 (9.3)	106 (12.2)	0.111
Everolimus, n (%)	0	0	n.a	0	0	n.a	0	0	n.a	0	5 (0.6)	0.095
Total number of drugs, median (quartiles) (5-95 perc)	2 (2–2)2 (2–3)	2 (2–2)2 (2–2)	0.019	3 (3–3)3 (2–3)	3 (3–3)3 (3–3)	0.019	3 (3–3)3 (2–3)	3 (3–3)3 (3–3)	0.017	3 (3-3)3 (2-3)	3 (3-3)3 (2-3)	0.122
Death or graft loss before discharge n (%)^c^	70 (15.0)	111 (14.8)	0.979	26 (6.2)	41 (6.0)	0.989	17 (4.9)	23 (3.8)	0.493	7 (1.4)	10 (1.1)	0.825
Protocol missing at discharge, n (%)	134 (28.6)	232 (31.0)	0.414	127 (30.0)	187 (27.3)	0.353	65 (18.8)	96 (15.7)	0.258	18 (3.5)	34 (3.7)	0.987

^a^ For 1198 patients no data were registered at discharge, and for 305 patients there was death or graft-loss before discharge - distribution presented in the bottom of the table.

^b^ Number of valid protocols at discharge. This number was used as the denominator for calculating the percentage of use of each drug.

^c^ Majority depending on graft-loss (50 patients did not survive until discharge - most of these during the two first periods).

n: number; n.a: not applicable (due small groups); p-value consider comparisons women vs. men; MMF: *mycophenolate mofetil*.

Due to revisions of the registry over time, some variables were added to the registry in later years. Comorbidity data (Supplementary Table 1), cold ischemia time, and biopsy register data (Table 3) were available only for patients with KT after 2007; therefore, an extended model including these variables was performed for the last time period. For the “comorbidity variable” only the main diagnosis representing “primary comorbidity” was registered for each patient; this evaluation was performed by the responsible physician. Biopsy data, procedures, and classification of the findings have been explained in a previous study [19].

**Table 3.** Distribution of biopsy^a^ findings among women and men kidney-transplanted from 2006 to 2017, *n* = 820.

Diagnosis at biopsy	Men, *n* = 533 n (Col%)	Women, *n* = 287 n (Col%)	*p*-value
Normal biopsy findings	47 (8.8%)	20 (7.0%)	0.430
Infections and tubulointerstitial nephritis (TIN)	28 (5.3%)	12 (4.2%)	0.610
Acute tubular injuries (ATN and acute CNI-toxicity)	93 (17.4%)	49 (17.1%)	0.969
Chronic changes including IFTA	102 (19.1%)	48 (16.7%)	0.449
Hematological diseases	3 (0.6%)	0 (0%)	0.505
Glomerular diseases	9 (1.7%)	4 (1.4%)	0.977
Minor abnormalities	80 (15.0%)	39 (13.6%)	0.655
Borderline changes	59 (11.1%)	19 (6.6%)	0.052
Rejections	112 (21.0%)	96 (33.4%)	<0.001

^a^ 94% of the analyzed biopsies were indication biopsies; Col%: column %; TIN: infections and tubulointerstitial nephritis; ATN: acute tubular necrosis; CNI: calcineurin inhibitor; IFTA: interstitial fibrosis and tubular atrophy; Glomerular diseases = recurrent or de-novo disease; Minor abnormalities were defined as minimal findings and none of the diagnosis above.

Rejection of a kidney graft should, according to a recent definition by Becker et al. be based on histopathological findings of phenotypes- borderline changes, T-cell or antibody-mediated acute or chronic changes, or their combinations [20]. In the present study, we used those criteria except for borderline changes that were kept as a separate group.

Cold ischemia time is defined as the span between the time point when the organ is removed from the donor and the time point when it is transplanted into the recipient [21].

### Immunosuppressive therapy (IST)

Number of patients who received induction therapy, according to sex and time period is shown in Supplementary Table 2.

Maintenance therapy primarily consists of a combination of immunosuppressive medications that target the immune system to prevent transplant rejection and graft loss. In recent decades, this treatment mostly consisted of a combination of calcineurin inhibitors (cyclosporine or tacrolimus), corticosteroids (prednisolone), and mycophenolate mofetil (MMF), whereas corticosteroids (prednisolone) and azathioprine were mostly used further back (Table 2). Kidney transplant patients were treated with the IST and were followed up according to the standardized immunosuppressive protocols recommended by the transplantation center. The therapy dose for each patient was adjusted according to the monitored drug concentration in the blood.

### Missing data

Donor-specific data were not available in the registry, such as panel-reactivity antibodies (PRA) or donor-specific antibodies (DSA), except for the time of ischemia and type of donor (living/deceased donor). Cold ischemia time was available only for the last transplantation period; in this period, 13% of the patients had missing data.

Patient characteristics in the registry did not include information about ethnicity, genetic factors, pregnancy, blood transfusions, and medical adherence (MNA). Follow-up data included the type of immunosuppression but did not include the drug dose.

The discharge protocol was missing for 19% of the patients (893 of 4698). However, the proportion of patients missing the discharge protocol decreased over time, that is, 30% for the period 1965–1985, 28% for 1986–1995, 17% for 1996–2005, and only 3.6% for 2006–2017; there were no differences in the amount of missing data between women and men (Table 2). Missing data were handled using missing categories when variables were included as covariates in the multivariable models.

### Statistical analysis

Background patient characteristics are presented separately for women and men and for different time periods as frequencies and percentages for categorical variables and as means and standard deviations, (SD) or medians and per­centiles for continuous variables. Corresponding group ­differences were formally tested using the χ^2^-test or Mann–Whitney test.

Time to event was defined as the time from the day of KT to the time of graft loss. The follow-up for each patient was censored at the time of death, the last follow-up (dropout), or the end of the study period (30 September 2017), whichever occurred first. Crude estimates of graft loss were presented using Kaplan-Meier survival curves. The log-rank test was used to compare the curves between women and men during the entire study period as well as for the four different time periods representing various IST regimes.

To adjust for potential confounding factors, we used multivariable Cox proportional hazard regression with time-to-graft loss as the outcome. Since the Kaplan-Meier curves indicated major deviations from the proportional hazard assumption, the Cox models were restricted to 10 years of follow-up, where no major violations of the PH assumptions were detected [22]. The time of death was used as a censoring point to account for the competing risk of death, and the outcome was primarily presented as cause-specific hazard ratios (HR) with 95% confidence intervals (CI). To further evaluate the effect of death as a competing risk, subdistributional hazard ratios (sHR) based on Fine JP & Gray RJ [23] were calculated for the main findings.

The *initial multivariable Cox model* included age, primary renal disease (PRD) diagnosis, donor type, and transplantation period as independent factors. To test whether sex-related differences changed over time, the model also included a sex-by-time period interaction. Following a significant sex-by-time period interaction, subsequent analyses were stratified by transplantation period.

An *extended multivariable Cox model* included all kidney transplant patients from 2006 to 2017 and all independent factors where the p-value was below 0.2 in the univariate Cox-model (Table 4). Additional factors that were available for patients transplanted between 2006–2017 were comorbidity, cold ischemia time, immunosuppressive therapy at discharge, and induction therapy.

**Figure 1.**
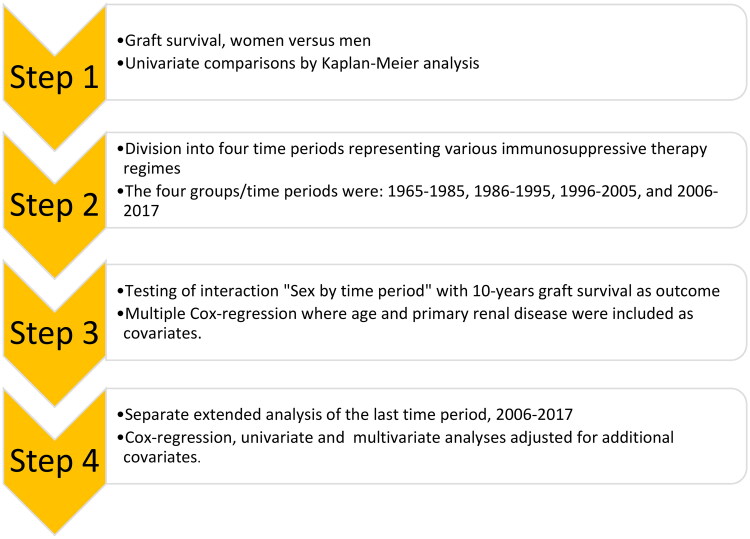
Illustration of the different steps of the performed data analyses and procedures.

For the last transplantation period, 58% (*n* = 820) of the patients had access to biopsy-based diagnoses, and comparisons were made between women and men (Table 3). The main analyses presented were based on our primary model, which was not adjusted for biopsy findings. However, a sensitivity analysis with biopsy findings was also performed (model 2 in Table 4). All steps of the statistical analyses are presented in Figure 1.

A result with a *p* < 0.05 was considered statistically significant if not otherwise mentioned.

IBM SPSS Statistics for Windows, Version 28.0 (Armonk, NY, USA) and Stata Statistical Software for Windows: Release 17 (College Station, TX, USA) were used for statistical analyses.

## Results

### Patient characteristics

In this study, 4698 patients (1742 (37%) women and 2956 (63%) men) who underwent kidney transplantation between 1965 and 2017 were analyzed, with graft survival as the outcome. For the entire group, the median age at transplantation was 48 years (quartiles 36–57 years). The median age was similar for women and men, but there were slight differences at different time periods (Table 1). During the period 1965–1985 the average annual number of transplantations was 61, for 1986–1995 it was 110, for 1996–2005 it was 96; and for 2006–2017 it was 118. The proportion of deceased donors did not differ between women and men during any of the transplantation periods (Table 1).

Women and men differed with respect to the distribution of PRD in each transplantation period (*p* < 0.001) (Table 1). Pyelonephritis was generally more common in the earlier time periods and less in the later time periods. Women had more pyelonephritis than men in the first three time periods (34% vs. 14% in the first period), whereas this difference disappeared in the last period (2.6% vs. 2.5%). Glomerulonephritis was generally more common in men than in women, and there was a general decrease over time (46% vs. 28% in the first period, and 29% vs. 18% in the last period) (Table 1).

Cold ischemia time was available only for the last transplantation period, and there was no difference between women (median= 9.4 h; interquartile range (IQR):1.9–14.4) and men (median = 9.8 h; IQR:2.1–14.3), *p* = 0.499.

Induction therapy was by methylprednisolone during all four periods but decreased slightly from above 99% during the two first periods to around 64% in the last period. Basiliximab was introduced in the period 1996–2005 (9% women, 6% men) and increased in the last period (62% women and 63% men). Rituximab was introduced in the last period and there was an observed sex-difference (14% among women and 8% among men, *p*-value < 0.001).

Maintenance therapy for almost all patients implied combinations of different drugs in all four time periods. Prednisolone was the only drug that was dominantly present in above 90% of the patients in all four time periods. During the first time period (years 1965–1985) the dominant drug was azathioprine (above 80%), during the second time period (years 1986–1995) the predominant drugs were cyclosporine (above 99% of the patients) and azathioprine (above 90% of the patients), during the third time period (years 1996–2005) predominant drugs were cyclosporine (above 79% of the patients) and MMF (above 62% of the patients) and during the last time period (years 2006–2017) predominant drugs were MMF (above 80%) and tacrolimus (62% among men and 54% among women) (Table 2).

### Graft survival – univariable analysis

**Figure 2.**
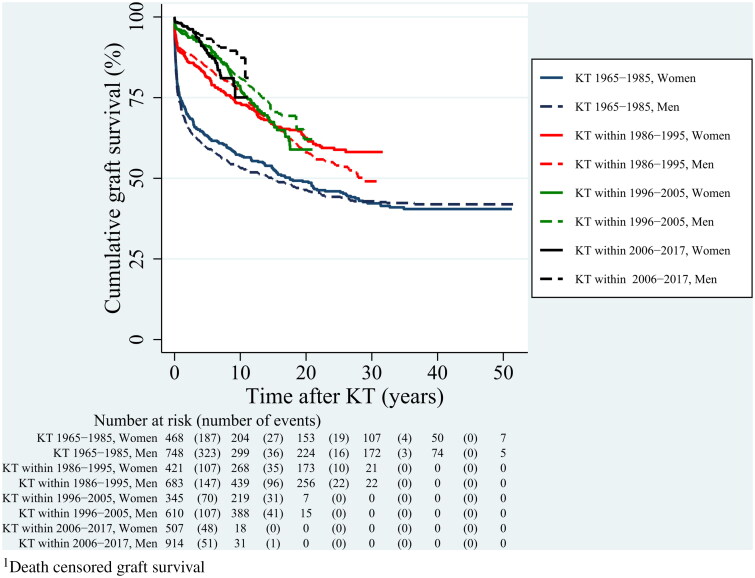
Graft survival^1^ after kidney transplantation (KT) in four time periods according to sex and during total observed follow-up. Kaplan-Meier curves.

**Figure 3.**
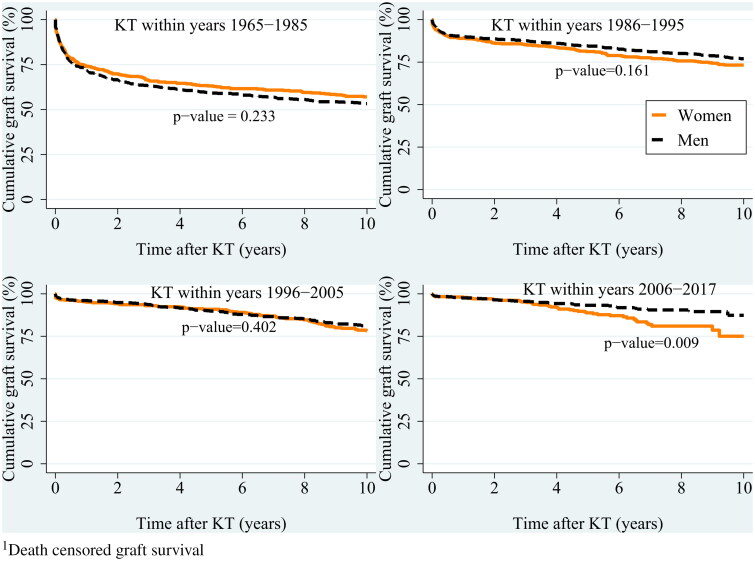
Comparison of 10-year graft survival^1^ between men and women stratified on four time periods of kidney transplantations (KT). Kaplan-Meier curves. P-values based on log-rank test.

During the observed study period, graft survival improved in both men and women (Figures 2 and 3).

The 5-years and 10-years overall graft survival rates were 61% and 55% for 1965–1985, 83% and 76% for 1986-1995, 90% and 80% for 1996-2005, and 92% and 83% for 2006–2017. In the last period, men had better graft survival than women, with a 5-year survival rate of 93% versus 89%, and a 10-year survival rate of 87% versus 75% (Figure 3, *p* = 0.009).

### Multivariable analysis of 10-years graft survival

After adjustment for age, primary renal disease, and donor type, the sex-related difference in graft survival 10 years after KT varied over time (sex-by-period interaction, *p* = 0.026).

Furthermore, for patients transplanted between 2006-2017, we performed an extended multivariate Cox regression where additional factors that were available only for this period were included (Table 4), which confirmed that women had shorter graft survival compared to men (HR = 1.71, 95% CI 1.1–2.7, *p* = 0.022).

Additionally, the Fine and Gray subdistribution hazard regression [23] adjusted for all confounders showed similar HRs as the cause-specific HRs (sHR:1.85, 95% CI:1.21–2.55, *p* = 0.005) for women versus men in the last transplantation period.

**Table 4.** Pre- and post-operative variables and their association with risk for graft loss for patients transplanted from 2006 to 2017.

Variable	HR with 95% CI, Univariate model	HR with 95% CI, Multiple model 1 (primary)	HR with 95% CI, Multiple model 2
Women^a^	1.68* (1.13–2.49)	1.71* (1.08–2.69)	1.69* (1.04–2.74)
Age (years)	0.99 (0.97–1.004)	0.99 (0.97–1.01)	0.99 (0.97–1.01)
Cold ischemia time (hours)	1.03 (0.99–1.06)	1.03 (0.98–1.09)	1.00 (0.95–1.06)
Deceased donor^b^	1.57* (1.01–2.43)	1.03 (0.48–2.25)	1.11 (0.48–2.56)
Primary renal diagnosis			
Glomerulonephritis/ sclerosis	Reference	Reference	Reference
Pyelonephritis	1.76 (0.68–4.60)	1.03 (0.30–3.45)	1.18 (0.34–4.1)
Polycystic kidney disease	0.58 (0.27–1.23)	0.49 (0.22–1.12)	0.40* (0.17–0.95)
Renal vascular disease due to hypertension hypertension	0.65 (0.15–2.76)	0.36 (0.05–2.72)	0.28 (0.04–2.09)
Renal vascular disease (unspecific)	2.62 (0.79–8.66)	3.0 (0.89–10.1)	1.82 (0.54–6.20)
Diabetes mellitus	0.78 (0.40–1.51)	0.62 (0.29–1.31)	0.53 (0.25–1.14)
Miscellaneous	1.01 (0.57–1.78)	0.72 (0.37–1.41)	0.49 (0.23–1.03)
Unknown	1.05 (0.58–1.91)	0.91 (0.47–1.77)	0.76 (0.38–1.53)
Comorbidity			
No comorbidity	Reference		
Diabetes mellitus	0.80 (0.29–2.23)	–	–
CVD	1.46 (0.66–3.24)	–	–
Hypertension	0.59 (0.08–4.27)	–	–
Endocrinological disease	n.a.	–	–
Pulmonal disease	0.75 (0.46–1.24)	–	–
Gastrointestinal and liver disease	1.35 (0.42–4.35)	–	–
Musculoskeletal disease	0.75 (0.10–5.43)	–	–
Neuro-psychiatric disease	0.81 (0.20–3.33)	–	–
Other diseases	1.20 (0.43–3.33)	–	–
Therapy/drugs at discharge			–
Prednisolone	1.31 (0.41–4.16)	–	–
Cyclosporine	1.09 (0.70–1.68)	–	–
Mycophenolate mofetil (MMF)	1.03 (0.62–1.69)	–	–
Tacrolimus	0.98 (0.64–1.51)	–	–
Myfortic	0.91 (0.53–1.56)	–	–
Tacrolimus depot	0.43 (0.10–1.75)	–	–
Total number of drugs	0.80 (0.48–1.32)	–	–
Induction therapy/drugs			–
Methylprednisolone	1.15 (0.67–1.95)	–	–
Anti-thymocyte globulin (ATG)	1.58 (0.72–3.49)	–	–
Basiliximab	1.05 (0.60–1.84)	–	–
Rituximab	0.61 (0.26–1.43)	–	–
Total number of ind. drugs	0.97 (0.76–1.25)	–	–
Diagnosis at biopsy			
Normal biopsy findings	Reference		Reference
Infections and TIN	1.24 (0.28–5.52)	–	1.39 (0.27–7.0)
Acute tubular injuries (ATN and acute CNI)	3.56* (1.25–10.18)	–	3.56* (1.05–12.1)
Chronic changes including IFTA	0.88 (0.28–2.78)	–	0.88 (0.23–3.37)
Hematological diseases	n.a	–	n.a.
Glomerular diseases	1.88 (0.34–10.29)	–	0.99 (0.10–9.87)
Minor abnormalities	1.11 (0.32–3.79)	–	1.06 (0.25–4.54)
Borderline changes	0.74 (0.19–2.98)	–	0.95 (0.21–4.38)
Rejections	2.12 (0.74–6.03)	–	1.85 (0.54–6.36)

TIN: Infections and tubulointerstitial nephritis; ATN: Acute tubular necrosis; CNI: Calcineurin inhibitor; IFTA: Interstitial fibrosis and tubular atrophy; Glomerular diseases: recurrent or de-novo disease; n.a: not applicable (small numbers). HR based on univariate and multiple Cox-regression model with 10-years graft survival as outcome. Minor abnormalities were defined as minimal findings and none of the diagnosis above; Multiple model 1 includes all variables with *p*-value < 0.2 from the univariate analysis except for biopsy findings; Multiple model 2 includes all variables with *p*-value < 0.2 from the univariate analysis.

^a^Women compared to men.

^b^Deceased donors compared to living donors.

*p-value < 0.05.

### Observed sex-differences in treatment and biopsy proven diagnosis

Table 2 shows that the IST prescribed at discharge after transplantation during the first three periods did not differ between women and men, whereas during the last period (2006–2017), a larger proportion of women compared to men received *tacrolimus* (62% vs 54%, *p* < 0.01) and *mycophenolate mofetil* (MMF, 85% vs 81%, *p* < 0.05). The total number of drugs did not differ between women and men (a median of 3 drugs for both women and men for 2006–2017).

Overall, induction therapy was similar among women and men. However, for the last transplantation period, some drugs, such as rituximab, were more common in women (14%) than in men (8%) (Supplementary Table 2).

Kidney transplant biopsies were performed in 820 patients, representing 58% of all patients transplanted between 2006–2017. The majority of biopsies were performed on clinical indications, for example, a decreased kidney function, increased proteinuria/hematuria, or other clinical symptoms indicating an impaired function of the kidney transplant. Protocol biopsies were only performed in clinical studies at our center and represented only around 6% of the biopsies in the study. Table 3 shows a more frequent prevalence of biopsy-proven rejections among women than among men (33% vs. 21%, *p* < 0.05). However, adjusting for this difference had no major effect on HR for sex, Table 4. We also explored the subtypes of rejection and found that differences between women and men were not dependent on a single subtype of rejection. Instead, we found that nearly all subtypes of rejection were slightly more common in women than in men (Supplementary Table 3).

## Discussion

The present study showed overall improved graft survival over time, as has been shown in other studies [4]. This is mainly due to changes in immunosuppressive therapy [4].

In analyzing the entire time period from 1965 to 2017, there was no difference in graft survival between women and men. However, when analyzing the different transplantation periods separately, we observed shorter graft survival among women than among men during the last transplantation period (2006–2017). This is in contrast to a study from Taiwan [15] in which women had better graft survival than men. It should be noted that the Taiwanese study included KT between 1988 and 2009, which was earlier than the last period in the present study. A recent study from Spain [18] showed lower graft survival for women under 60 years of age, which is more consistent with the present study.

During the last period of the present study, we found a higher proportion of rejection among women than men. However, as the difference in graft survival remained after adjustment for biopsy-based diagnosis in a multivariable model (Table 4) there must be other explanations. Lowest graft survival was observed in transplants where biopsies detected acute tubular injuries. However, in an earlier study of the same registry data [19] where the date of biopsy was used as a baseline instead of the date of transplantation, the lowest graft survival was observed in glomerular diseases and rejections.

Medication non-adherence (MNA) is one of the most important causes of shortened graft survival and kidney rejection [24–28]. A risk group for MNA appears to be kidney recipients with lower perceived social support, lower mental and physical health-related quality of life, and younger age [29]. Some studies have shown favorable MNA-outcome in female kidney transplant recipients [24,30]. In the present study, no data were available regarding MNA.

Over the analyzed time periods, we noted an increase in the mean age of patients. Age is known to have an impact on graft loss [11], there was no difference in age between women and men in our study. In addition, overall age was not associated with graft loss. However, older age may discriminate men from women, since older women would be more exposed to previous pregnancies than younger women. However, studies have shown that post-transplant pregnancy does not affect the risk of graft failure [31–33]. On the other hand, pre-transplant history of pregnancy may be associated with higher rates of rejection owing to higher panel reactive antibody levels [5]. This could have contributed to the worse outcomes among women in the present study, and if so, this might mean that women require more intensive IST. However, in the present study, we had no data regarding panel-reactivity antibodies and neither of the levels of chosen doses (low risk vs. high risk) of IST.

Another reason could be frailty among women with advanced chronic kidney disease, which in a previous study was twice as frequent as in men and was mostly associated with poorer results in socioeconomic variables [34]. The present study lacked information regarding frailty. Other preoperative factors associated with graft survival are donor and recipient characteristics such as age, race, sex, immunologic compatibility, and some preoperative and postoperative factors such as ischemia time, IST, and IST dose [5]. However, several of these variables were included as confounders in our model.

Notably, in the present study, a difference in graft survival between women and men was observed only during the last transplantation period. This may be due to new ISTs, such as mycophenolate mofetil (MMF) and calcineurin inhibitors (tacrolimus or cyclosporine), which have been introduced in recent decades. There were higher proportions of MMF and tacrolimus use among women. MMF and mTOR inhibitors (sirolimus or everolimus) have been associated with higher rates of acute rejection [35], but this was not the case in the present study.

Strengths of our study were the cohort design that included a large number of kidney-transplanted patients who were followed up for a long time after transplantation and access to many possible covariates, particularly in the last transplantation period where a sex difference was observed.

The analyzed graft survival data were based on many follow-ups and the extended time after KT, which made it possible to detect and explore differences that might have been missed or underestimated in other studies.

The coverage of the transplantation registry is estimated to be 80–90% and selection bias is unlikely to have had a meaningful effect on the main findings.

For sub-analysis of biopsy data, the possibility of selection bias needs to be considered. As biopsies were performed on the indication, the data may not fully represent the entire transplanted group of patients.

Medication (IST) was measured based on the discharge protocol, which may not reflect compliance and change in treatment later on for each patient. Drug concentration was not registered in the transplantation registry; therefore, we could not include it in our model, but all patients were prescribed the IST according to the standardized immunosuppressive protocols recommended by the transplantation center.

There were missing protocols and information regarding treatment at discharge for 19% of the patients; however, the proportion was similar in men and women and was only significantly different for the last transplantation period (3.5% for women and 3.7% for men).

Finally, unmeasured confounding factors affecting the findings, such as ethnicity, genetic factors, pregnancy, organ quality, blood transfusions, or donor-specific characteristics, cannot be precluded. In addition, no donor-specific data were available in the registry because registration of such data was missing in the registry.

However, we would expect HLA matches (HLA = human leukocyte antigens) to be similar between men and women while eventually other antibodies based on pregnancies may differ. Since transfusions are rare in general, we think this variable is of less importance.

In conclusion, the present study showed that women had shorter graft survival than men during the last observation period (2006–2017). Biopsy-proven rejection was more common in women. It cannot be precluded that these results may be related to new immunosuppressive therapies such as mycophenolate mofetil (MMF) and a calcineurin inhibitor (tacrolimus or cyclosporine), which have been introduced in recent decades. There are likely several factors involved in the explanation of higher graft-loss rates among women. Further studies are warranted to confirm the shorter graft survival among women in the last study period and to investigate the factors that have an impact on graft survival.

## Supplementary Material

Supplemental Material

## Data Availability

The data that support the findings of this study are available upon reasonable request from the corresponding author. The data are not publicly available because of regulations or ethical restrictions.
