# Supplementary material for: Sex-specific time trends of long-term graft survival after kidney transplantation – a registry-based study
Source: Ren Fail. 2023 Oct 26;45(2):2270078. doi: 10.1080/0886022X.2023.2270078 (PMC11001319; doi:10.1080/0886022X.2023.2270078)
Supplement: Supplemental Material [file IRNF_A_2270078_SM3345.pdf]

SUPPLEMENTARY MATERIAL

Supplementary Figure 1. Graft survival[1] after all kidney transplantations from 1965 to 2017 (n=4698). Kaplan-Meier curves.

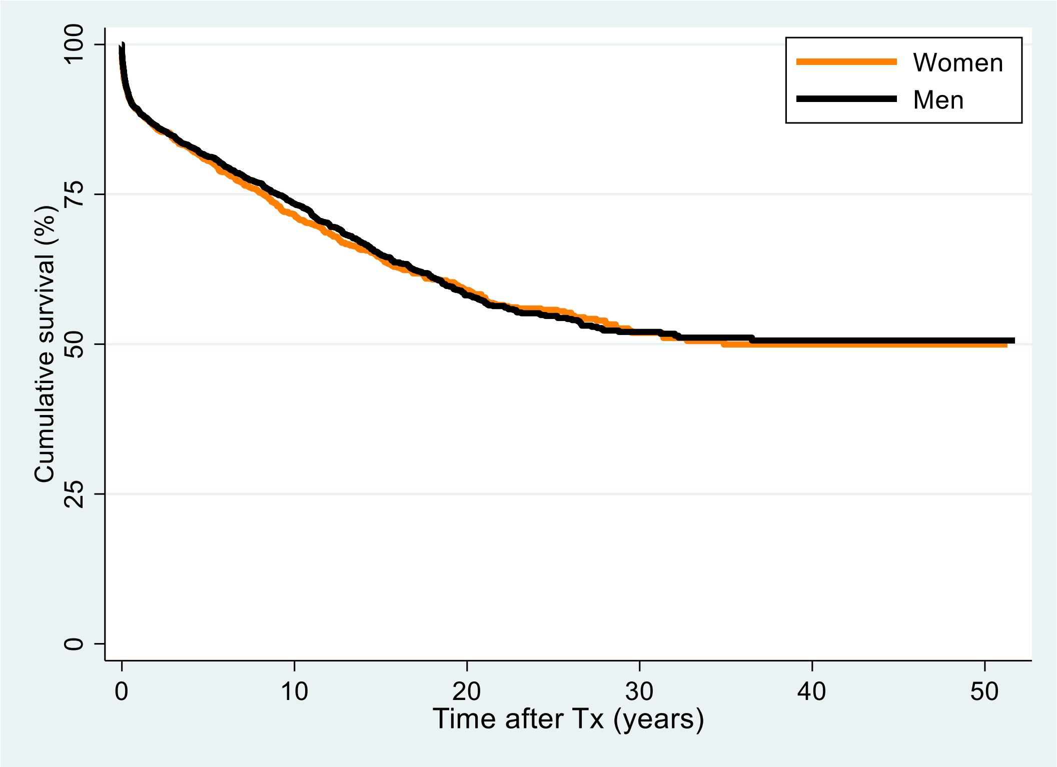

[1] Death censored graft survival; p-value=0.409, Log-rank test.

Supplementary table 1: Comorbidity[1] distribution among men and women who were kidney-transplanted from 2006 to 2017.

**Comorbidity**	**Women n (Col%)**	**Men n (Col%)**
No comorbidity	158 (31.2%)	239 (26.1%)
Diabetes mellitus	24 (4.7%)	40 (4.4%)
Cardiovascular disease (CVD)	10 (2.0%)	50 (5.5%)
Hypertension	246 (48.5%)	479 (52.4%)
Endocrinological disease	14 (2.8%)	8 (0.9%)
Pulmonal disease	6 (1.2%)	14 (1.5%)
Gastrointestinal and liver disease	8 (1.6%)	19 (2.1%)
Musculoskeletal disease	9 (1.8%)	7 (0.8%)
Neuro-psychiatric disease	14 (2.8%)	21 (2.3%)
Other diseases	18 (3.6%)	37 (4.0%)
Total n	**507**	**914**

[1]Comorbidity data were registered only in the last transplantation period 2006-2017 and with only one comorbidity diagnosis per patient. Col%= column %; Chi-square test, p-value=0.003

Supplementary table 2: Induction therapy among women and men. Based on protocols registered at discharge after transplantation. Occurrence (%) of each drug. 3500 of 4698 transplanted patients had valid protocols at discharge[a].

	Time period for transplantation (years)							
	1965-1985		1986-1995		1996-2005		2006-2017	
Total number	Women n=468	Men n=748	Women n=421	Men n=683	Women n=346	Men n=611	Women n= 507	Men n=914
Valid protocols[b]	n=264	n=405	n=268	n=455	n=264	n=492	n=482	n=870
Methylprednisolone, n (%)	263 (99.6)	401 (99.0)	266 (99.3)	451 (99.1)	246 (93.2)	462 (93.9)	307 (63.7)	561 (64.5)
Anti-thymocyte globulin (ATG), n (%)	4 (1.5)	2 (0.5)	56 (20.9)	82 (18.0)	42 (15.9)	92 (18.7)	45 (9.3)	87 (10.0)
Basiliximab, n(%)	0	0	0	0	25 (9.5)	28 (5.7)	298 (61.8)	547 (62.9)
Rituximab, n (%)	0	0	0	0	1 (0.4)	0	68** (14.1)	71 (8.2)
Daclizumab, n (%)	0	0	1 (0.4)	0	20 (7.6)	24 (4.9)	8 (1.7)	8 (0.9)
OKT3, n (%)	0	0	0	1 (0.2)	1 (0.4)	11 (2.2)	1 (0.2)	0
Unspecific drug, n (%)	2 (0.8)	5 (0.3)	18* (6.7)	18 (4.0)	9 (3.4)	25 (5.1)	108 (22.4)	188 (21.6)
Total number of drugs, median (quartiles) (5-95 perc)	1 (1-1) 1 (1-1)	1 (1-1) 1 (1-1)	1 (1-2) 1 (1-2)	1 (1-1) 1 (1-2)	1 (1-2) 1 (1-2)	1 (1-2) 1 (1-2)	2 (1-2) 2 (0-3)	2 (1-2) 2 (0-3)
Death or graft loss before discharge.[c]	70 (15.0)	111 (14.8)	26 (6.2)	41 (6.0)	17 (4.9)	23 (3.8)	7 (1.4)	10 (1.1)
Protocol missing at discharge	134 (28.6)	232 (31.0)	127 (30.0)	187 (27.3)	65 (18.8)	96 (15.7)	18 (3.5)	34 (3.7)

[a] For 1198 patients no data at discharge was registered, and for 305 patients there was death or graft-loss before the discharge distribution presented in the table above.

[b] Number of valid protocols at discharge. This number was used as the denominator for calculating percentage of use of each drug.

[c] Majority depending on graft-loss (50 patients did not survive until discharge- most of these during the two first periods).

n=number; n.s = not significant; * p-value<0.05 women vs. men; ** p-value <0.001 women vs men; MMF=mycophenolate mofetil.

Supplementary Table 3: Distribution of subgroups of rejections among women and men. Based on biopsy-proven rejections (n=208).

	**Women n=96**	**Men n=112**
	**n (Col%)**	**n (Col%)**
**Acute TCMR (n=165)**	70 (72.9%)	95 (84.8%)
**Chronic TCMR (n=3)**	1 (1.0%)	2 (1.8%)
**Active ABMR (n=12)**	8 (8.3%)	4 (3.6%)
**Chronic ABMR (n=10)**	6 (6.3%)	4 (3.6%)
**Combined active ABMR and acute TCMR (n=3)**	3 (3.1%)	0 (0%)
**Combined chronic ABMR and chronic TCMR (n=15)**	8 (8.3%)	7 (6.3%)

No statistically significant difference between men and women, p-value=0.171. Col%= column %.

TCMR=T-cell mediated rejection; ABMR=antibody-mediated rejection.
